# Clinical use of autologous cell-based therapies in an evolving regulatory landscape: A survey of patient experiences and perceptions

**DOI:** 10.12688/f1000research.141002.1

**Published:** 2023-09-18

**Authors:** Ubaka Ogbogu, Nevicia Case

**Affiliations:** 1 Faculty of Law, University of Alberta, Edmonton, Alberta, T6G2H5, Canada

**Keywords:** autologous cell-based therapy, cell therapy, stem cells, advanced therapies, regenerative medicines, health product regulation, patient experience, patient education, stem cell regulation, clinical trial ethics, pay-to-participate trials, healthcare providers

## Abstract

**Background**: Clinical treatments involving autologous cell-based therapies (ACBT) remain prevalent despite a lack of scientific backing and an evolving regulatory landscape aimed at assessing their safety and efficacy for clinical adoption. This study seeks to assess patients’ experiences and perceptions of clinical treatments involving ACBT and their knowledge and views of the regulatory context and associated governance issues.

**Methods**: An anonymous online survey of 181 participants who have been treated or are in the process of being treated with ACBT was conducted. Recruitment was via social media platforms. Data was collected through Qualtrics and analyzed using SPSS 29 for the quantitative responses and NVivo 1.7.1 for the qualitative responses.

**Results**: Several themes emerged from the data, including the prominent role of healthcare providers throughout the patient journey, informational practices during the clinical encounter, the high prevalence of pay-for-participation trials, patients’ gaps in regulatory knowledge, and patients’ priorities regarding clinical trials and regulation of ACBT.

**Conclusions**: The study makes a novel contribution to the literature by providing the first analysis of patients’ experiences and perceptions of an emerging cell-based therapy within an evolving regulatory landscape. The findings serve as a valuable resource for developing policy, promoting scientific rigor, and ensuring ethical oversight of ACBT and other upcoming cell-based therapies.

## Introduction

It has been widely reported in the literature and news media that individuals are seeking and obtaining putative cell-based therapies in advance of regulatory approvals and, in many instances, prior to the availability of any or high-quality evidence ^1^^,^^2^ supporting their safety, effectiveness and/or clinical adoption. ^3^ The first study of this phenomenon, published in 2005, described stem cell-based therapies with little or no evidentiary support offered mainly via online marketing to patients who were willing to travel internationally to receive them. ^4^ Since then, the phenomenon has evolved into a national and international industry in many jurisdictions, including in countries with highly developed health products regulatory systems, such as Canada, Australia, the European Union, and the United States (U.S.). ^5^^–^^7^ Studies have tracked the proliferation of treatment providers in countries around the world serving local and international clientele, ^8^ using a variety of strategies to reach potential clients, ^9^ and offering a variety of interventions that are often neither approved by regulators, nor backed by high quality evidence. ^10^ Studies have also reported on the adverse events associated with these interventions, ^11^ the experiences of patients who access the treatments, and their reasons for doing so, ^11^^,^^12^ provider perspectives, ^13^ and the evolution of and trends in the industry. ^14^

The phenomenon has attracted regulatory, legal, ethical and media scrutiny and responses, including sanction by regulators, ^15^ court challenges, ^16^ legislative and policy reform, ^17^ and policy recommendations addressing associated issues and challenges, such as the role of consumer protection and truthful advertising in addressing false therapeutic claims, ^18^ and healthcare professional involvement and ethics. ^19^ Some jurisdictions have established or are in the process of establishing legal and regulatory changes designed to clarify and strengthen the framework for regulatory review and marketing authorization of regenerative medicines, which include cell-based therapies. ^15^^,^^19^^,^^20^ These changes respond, in part, to legitimate questions about how to classify and assess these therapies, many of which are innovative and present differently from conventional pharmaceuticals. ^21^ Regulatory difficulties also arise from claims, usually made by treatment providers that certain cell-based therapies are not within regulatory scope because they are not health products per se, but, rather, are simply medical procedures that utilize cells or cell-based products. ^22^^–^^24^

The latter claim is most evident in the regulatory debate surrounding the use of human-derived autologous cells in medical procedures, which is often referred to as “autologous cell-based therapy” (ACBT). Generally, ACBT “involves the removal, some level of manipulation or processing, and re-introduction of a person’s own cells to treat or prevent a disease, disorder or medical condition.” ^25^ There is mounting evidence of providers applying ACBT for the clinical management of a variety of medical conditions, and especially for osteoarthritis, musculoskeletal disorders, and sports injuries. ^26^ Jurisdictions such as the U.S., the European Union, Canada and Australia have sought to clarify whether and what cells used in ACBT constitute regulated health products. ^27^ While the details as to what constitutes a regulated “ACBT product” (i.e., cell or cell-derived material used in ACBT) varies by jurisdiction, some commonalities exist. Generally, some ACBT products are exempt from health product regulation (under certain conditions), while others require full pre-marketing approval under an appropriate regulatory pathway. ^17^^,^^28^^,^^29^ The differences between both categories depends largely on risk classification and in some cases, on whether and to what extent the cells have been manipulated prior to clinical administration. ^16^^,^^30^^,^^31^

However, this increased clarity appears not to have prevented the use of regulated but unapproved and/or unauthorized ACBT products in medical procedures, and there is evidence that patients continue to access such products. ^31^^–^^33^ As part of efforts to achieve regulatory compliance, some regulators, such as Health Canada and the Australian Therapeutic Goods Administration, have required treatment providers to conduct clinical trials to obtain evidence to support clinical deployment of various ACBT products. ^17^^,^^34^^,^^35^ This requirement has led to a proliferation of “pay-to-participate” or “pay-to-play” clinical trials, whereby patients seeking ACBT are asked to pay to participate in the trials. ^36^^,^^37^ There is also speculation that patients who have paid for what they think is a medical procedure are unwittingly enrolled in what is actually a clinical trial. ^38^ Both practices, to the extent that they exist, raise serious ethical concerns regarding informed consent, therapeutic misconception (mistaking participation in research as receiving clinical therapy and benefit), and exploitation of vulnerable patients. ^39^^,^^40^

In this study, we sought to assess patients’ experiences with and attitudes towards ACBT, and their knowledge regarding associated legal and regulatory matters. The study seeks to provide evidence regarding how and why patients are accessing innovative regenerative medicines in a context that is fraught with scientific, legal, ethical and regulatory uncertainties, questions, and concerns.

The study advances and makes two important contributions to the existing literature on questions surrounding the clinical use of cell-based therapy products in the absence of regulatory authorization or high-quality evidentiary support. First, it is the earliest known examination of patient perspectives regarding cell-based therapy products that are *within* regulatory scope, in the sense that regulators have defined parameters for their authorization and use for clinical research and/or clinical management of medical conditions. Second, the study offers insights on how patients are experiencing the impacts of emerging regulatory frameworks for ACBT products and cell-based therapy products more generally. Both contributions provide a basis for analyzing associated ethical, legal and regulatory concerns, including concerns regarding pay-to-participate trials, informed consent, therapeutic misconception, and regulatory compliance. The study also provides data that different policymaking bodies, including governments, scientific bodies, and health professions regulators, can rely on to develop policies aimed at addressing ethical and regulatory compliance issues, and at improving the clinical experience of patients accessing ACBT.

## Methods

### Ethics and consent

Ethics approval for this study was granted by the University of Alberta’s Research Ethics Board (Pro00128173). Participants provided full and voluntary consent to participate in the study and to the publication of the anonymous study results via a consent form approved by the University of Alberta’s Research Ethics Board. Participants read the consent form prior to completing the survey, and per the terms of the approved consent form, their consent to participate in the study was implied from the completion of the survey.

### Participants

The study participants included individuals who received or were in the process of receiving treatments involving ACBT. Recruitment was conducted through posts on social media platforms, including Facebook, Twitter, LinkedIn, and Reddit.

### Survey instrument, administration and response rate

The survey instrument explored participants’ knowledge about, perspectives of, and experiences with clinical procedures involving ACBT. The survey included a mix of questions eliciting quantitative and qualitative responses and took approximately 10 minutes to complete. The survey instrument is reproduced in the extended data. ^48^

The survey was administered via Qualtrics. Data collection occurred for the duration of 11 days (06-17 March 2023). The survey was closed when the response rate reached what we considered optimal for analysis and no new responses had been received for two days. Following data collection, the initial dataset, consisting of 347 responses, was cleaned for analysis. Responses marked by Qualtrics as duplicates ( *n* = 22), as likely bots ( *n* = 101), and as both duplicates and likely bots ( *n* = 5) were deleted from the dataset. Through manual data cleaning, any responses which were completely blank ( *n* = 18) or had multiple nonsensical text entries were categorised as likely bots ( *n* = 2) and were deleted from the dataset. We added a screening variable to ensure that participants had received ACBT. Seventeen participants indicated that the treatment they received did not involve taking cells, blood, or tissue from their own body, and one participant did not respond to the variable. We excluded these 18 participants based on the non-response or because it is likely they did not receive ACBT. The final dataset used for our analysis consisted of 181 responses. Among the 181 responses, missing data accounted for 1.3% of the values. As the percentage of missing values was below the 5% threshold that would likely affect the integrity of the dataset, ^41^ they were ignored in the analysis. For items where participants were asked to “check all that apply”, the total number of responses is reported. The final dataset is available in underlying data. ^48^

### Data analysis

The quantitative survey data were analysed using SPSS 29. Chi-square tests were used to identify patterns in the quantitative data. Qualitative survey responses were analysed by identifying themes emerging from the data using NVivo 1.7.1. The data analyses were not pre-registered.

## Results

### Part 1: Summary of participants’ experiences

Participants were asked to report what type of ACBT they received, how they learned about it, where and in what facility they received it, which type of treatment provider administered it, why and for what condition they sought it, whether the ACBT was combined with another product, whether it was presented as a clinical trial or as a medical procedure, and whether there were any side effects. They were also asked about payment, including whether and how much they paid for the ACBT, and if and how they were reimbursed for the amount paid, and about whether their treatment provider discussed details with them concerning the purpose of cells being removed from their body and side effects from the ACBT.

Of 181 participants, 53 (29.3%) reported that they received an autologous fat transplant, 48 (26.5%) received autologous human fibroblasts, 41 (22.7%) received an autologous serum therapy, 25 (13.8%) received autologous mesenchymal stem cells, and 14 (7.7%) received platelet-rich plasma. Of the 180 participants who responded to the variable regarding how they learned about the ACBT, 78 (43.3%) reported that they learned about the ACBT from the treatment provider, 47 (26.1%) were referred by a physician or clinic other than the treatment provider **,** 19 (10.6%) learned about it through social media, 16 (8.9%) received a referral from another healthcare provider (other than a physician or clinic), 12 (6.7%) learned about it through a clinic website, 4 (2.2%) through advertisements in a clinic, and 4 (2.2%) by word of mouth. Of 181 participants, public hospitals were the most common treatment facility (42.0%, *n* = 76), followed by specialists’ clinics (28.7%, *n* = 52), private hospitals (13.3%, *n* = 24), family doctors’ clinics (9.9%, *n* = 18), and walk-in clinics (6.1%, *n* = 11). Of 179 participants, most reported that their treatment provider was a doctor (89.9%, *n* = 161), while others reported that their treatment provider was a nurse practitioner (7.8%, *n* = 14), a nurse (1.7%, *n* = 3), or a physician assistant (0.6%, *n* = 1). Among 176 of the participants whose treatment provider was a doctor, their specialty was either in orthopaedics (46.0%, n = 81), sports medicine (31.8%, *n* = 56), plastic surgery (20.5%, *n* = 36), oncology (0.6%, *n* = 1), physiatry (0.6%, *n* = 1), or chiropractic medicine (0.5%, *n* = 1). Of 178 participants who responded, the majority received the ACBT in the U.S. (74.7%, *n* = 133), while the remainder received it in Canada (25.3%, *n* = 45). When asked to select all that apply, out of 260 selected responses, the reasons for seeking the ACBT varied with 106 (40.8%) participants reporting that their doctor or other healthcare provider recommended it, 82 (31.5%) indicated that they tried other treatments and they didn’t work, 39 (15.0%) indicated it was their only treatment option, 32 (12.3%) reported that a friend or family member recommended it, and 1 (0.4%) indicated that patients with their condition recommended it. Of 180 participants, the majority sought the ACBT for a sports injury (47.2%, *n* = 85) or osteoarthritis (36.7%, *n* = 66), while others sought it for cosmetic reasons (12.2%, *n* = 22) or other conditions (3.9%, *n* = 7). Three-quarters (76.2%, *n* = 138) of 181 participants reported that the ACBT was combined with another product, such as a medical device. The remainder of participants did not have an ACBT that was combined with another product (21.0%, *n* = 38) or were unsure (2.8%, *n* = 5).

Of 181 participants, most reported that the ACBT was presented as a medical procedure (70.2%, *n* = 127), 45 (24.9%) participants reported that it was presented as a clinical trial, and 9 (5.0%) participants were unsure. Of 181 participants, 170 (93.9%) reported that the purpose of removing the cells, blood, or tissue from their body was explained to them, while 11 (6.1%) indicated that the purpose was not explained. Of 181 participants, while most did not report experiencing any side effects from the ACBT (60.2%, *n* = 109), 72 (39.8%) reported that they experienced side effects. Of 181 participants, the majority (86.7%, *n* = 157) indicated that the treatment provider informed them about any possible side effects. The remainder of participants either indicated that the treatment provider did not inform them (3.9%, *n* = 7) or that they didn’t remember (9.4%, *n* = 17).

Of 181 participants, the vast majority reported paying for the ACBT (92.8%, *n* = 168), while 10 (5.5%) reported that they didn’t pay, and 3 (1.7%) didn’t remember. Among all of the participants who paid, most paid $100-$499 (39.9%, *n* = 67) or $500-$999 (32.7%, *n* = 55), while 37 (22.0%) paid $1000 or more. Only 9 (5.4%) paid $99 or less. All amounts were reported in Canadian Dollars. Among all of the participants who paid, 61 (36.3%) were reimbursed and 74 (44.0%) paid out-of-pocket. Thirty-three participants (19.6%) reported both being reimbursed and paying out-of-pocket. Out of 132 selected responses among those who received some degree of reimbursement, when asked to select all that apply, 61 (46.2%) participants indicated that they were reimbursed by government (public) health insurance, while 50 (37.9%) were reimbursed by private insurance through their employer, and 21 (15.9%) were reimbursed by private insurance not through their employer.

### Part 2: Knowledge of regulation of the treatment

Participants indicated their knowledge of who the ACBT is regulated by and the evidence for the ACBT. They also reported whether their treatment provider discussed these details with them. Participants rated their confidence in knowing who to contact if they would like to make a complaint regarding the ACBT and shared their knowledge of who to contact.

Of 176 participants, most indicated that the treatment provider discussed with them how the ACBT is regulated (88.6%, *n* = 156), while 8 (4.5%) participants indicated that it was not discussed, and 12 (6.8%) participants didn’t remember. When asked to select all that apply, out of 360 selected responses, 89 (24.7%) participants indicated that, to the best of their knowledge, the ACBT is regulated by a provincial medical regulatory authority, 88 (24.4%) indicated a provincial ministry of health, 77 (21.4%) indicated a federal regulatory authority, 41 (11.4%) indicated a federal medical regulatory authority, 31 (8.6%) indicated a research ethics board, 21 (5.8%) were unsure, and 13 (3.6%) indicated that there is no regulatory authority. In the event that they would like to make a complaint regarding the ACBT, 170 participants reported an average of 70.2% ( *SD* = 21.2) confidence in knowing who to contact. When asked to select all that apply, out of 361 selected responses, most participants indicated that they would contact the clinic where they received the treatment procedure (37.4%, *n* = 135), 100 (27.7%) indicated a government regulator, 75 (20.8%) a research ethics board, and 51 (14.1%) the professional regulator. Of 180 participants, while 165 (91.7%) indicated that the treatment provider explained to them the evidence for the ACBT, 7 (3.9%) indicated that they did not, and 8 (4.4%) didn’t remember whether or not this was explained. When asked to select all that apply, to the best of their knowledge, out of 438 selected responses, 121 (27.6%) participants indicated that the evidence for the ACBT was the treatment provider’s clinical experience, 111 (25.3%) indicated peer-reviewed scientific research, 97 (22.1%) indicated patient testimonials, 64 (14.6%) indicated randomised controlled trials, and 45 (10.3%) indicated regulatory approval.

### Part 3: Participants’ perceptions of the clinical encounter

Participants reported whether they thought the ACBT should be provided without going through clinical trials or regulation and why or why not. They indicated whether they would recommend the ACBT and treatment procedure to someone else. Participants were asked whether and how their perception of the ACBT would change if they were to learn that it did not go through clinical trials and was not approved by a regulator.

Of 179 participants, 74 (41.3%) indicated that they thought the ACBT should be provided without going through clinical trials or regulation and 105 (58.7%) disagreed. Participants provided 81 unique short answer text responses that expanded on their views. Sixteen of the participants who indicated that the ACBT should be provided without going through clinical trials or regulation provided reasons that were coded as relating to their beliefs (62.5%, *n* = 10), feelings (18.8% *n* = 3), subjective experiences (12.5% *n* = 2), or perspectives (6.3% *n* = 1). Among participants who shared their beliefs, 4 participants had beliefs regarding clinical trials, including the possibility of being assigned to a control group if they participate in a clinical trial and concerns about missing the best time for treatment given the lengthiness of clinical trials and regulatory approval. The remaining 6 participants were divided regarding whether they believed safety was a concern for ACBT. One participant expressed their feelings around the lack of safety, while two participants did not feel as though there is anything wrong with the ACBT. Two participants shared subjective experiences of the ACBT having helped them and one shared a perspective of wanting to give it a try regardless of undergoing clinical trials or regulatory approval.

Of the participants who thought that the ACBT should not be provided without going through clinical trials or regulation, 57 shared reasons that were coded as relating to their beliefs (77.2%, *n* = 44), feelings (17.5%, *n* = 10), perspectives (3.5%, *n* = 2), or subjective experience (1.8%, *n* = 1). Safety was their primary concern. Participants mentioned safety both with regards to their beliefs about the role of clinical trials and regulation in determining safety ( *n* = 23) and with regards to their subjective feelings of security and reassurance ( *n* = 10). Participants also expressed that their responses stemmed from their beliefs regarding the professionalism of healthcare providers in upholding the rigour of medicine ( *n* = 13) and the importance of regulation for ensuring accountability and reliability ( *n* = 4). The remaining 4 participants shared beliefs regarding the role of clinical trials in ensuring efficacy, systemic responsibility for people’s health, and a belief that the technology is immature. Two participants shared their perspectives of not wanting to be tested on and one participant mentioned a subjective experience of pain.

Of 180 participants, most indicated that they would recommend the ACBT to someone else (77.8%, *n* = 140), while 40 participants (22.2%) indicated that they would not recommend it.

Of 180 participants, the majority indicated that if they were to learn that the ACBT they received did not go through clinical trials and was not approved by a regulator, it would change their perception of the ACBT (85.6%, *n* = 154), while the remainder (14.4%, *n* = 26) indicated that it would not change their perception of the ACBT. Of these participants, 85 followed up with unique short answer text responses to share how their perceptions would change or not change. Among 67 of the participants who indicated that their perception would change, their reasons were coded to be rooted in their beliefs (50.7%, *n* = 34), feelings (28.4%, *n* = 19), perspectives (14.9%, *n* = 10) or subjective experiences (6.0%, *n* = 4). Participants shared their beliefs regarding safety concerns ( *n* = 18) and the lack of other treatments for their condition ( *n* = 6). The remaining 10 participants shared beliefs of uncertainty about the efficacy of ACBT and high risk in the absence of regulation and in the absence of clinical trials. Participants expressed their feelings of uncertainty regarding ACBT safety ( *n* = 15) and concerns around efficacy ( *n* = 4). Ten participants shared their perspectives regarding hesitation to receive ACBT and four participants based their response on their subjective experiences, including having tried many other treatments or the ACBT being the only available treatment option.

Among 13 of the participants who indicated that their perception would not change, their reasons were coded as relating to their beliefs (38.5%, *n* = 5), perspectives (23.1%, *n* = 3), awareness (15.4%, *n* = 2), subjective experiences (15.4%, *n* = 2), or feelings (7.7%, *n* = 1). Participants’ beliefs included the lack of other options and were divided regarding the safety of the ACBT. The remainder of participants expressed their perspectives regarding the risk of missing the best time for treatment, their subjective experiences of having tried other treatments that were unsuccessful, and the efficacy of the ACBT.

### Association between study variables depending on whether participants reported involvement in a clinical trial versus a medical procedure

Chi-square analyses identified statistically significant associations between several variables and the variable regarding whether participants received the ACBT as part of a clinical trial or a medical procedure. The facility where participants received the treatment procedure varied depending on whether they indicated receiving it as part of a clinical trial, or as a medical procedure, or were unsure, *X* ^2^ (8, 181) = 39.73, *p* < .001. Among participants who reported receiving the ACBT as part of a clinical trial, 18 (40.0%) reported received it in a specialist’s clinic, 11 (24.4%) in a family doctor’s clinic, 6 (13.3%) in a public hospital, 5 (11.1%) in a private hospital, and 5 (11.1%) in a walk-in clinic. Participants who reported receiving the ACBT as a medical procedure were most likely to receive it in a public hospital (53.5%, *n* = 68), followed by 32 (25.2%) in a specialist’s clinic, 16 (12.3%) in a private hospital, 7 (5.5%) in a family doctor’s clinic, and 4 (3.1%) in a walk-in clinic.

Participants who received the ACBT as part of a clinical trial or as a medical procedure varied in whether the treatment provider discussed with them how the ACBT is regulated, χ ^2^ (4, 176) = 10.80, *p* = .029. The majority of participants who indicated that they received the ACBT as part of a clinical trial reported having this information discussed with them (86.4%, *n* = 38), while 5 (11.4%) did not have this information discussed with them and 1 (2.3%) did not remember whether or not it was discussed. Participants who indicated that they received the ACBT as a medical procedure and reported having this information discussed with them were also in the majority (90.2%, *n* = 111), while 3 (2.4%) did not have this information discussed with them and 9 (7.3%) did not remember whether or not it was discussed.

**Figure 1.**
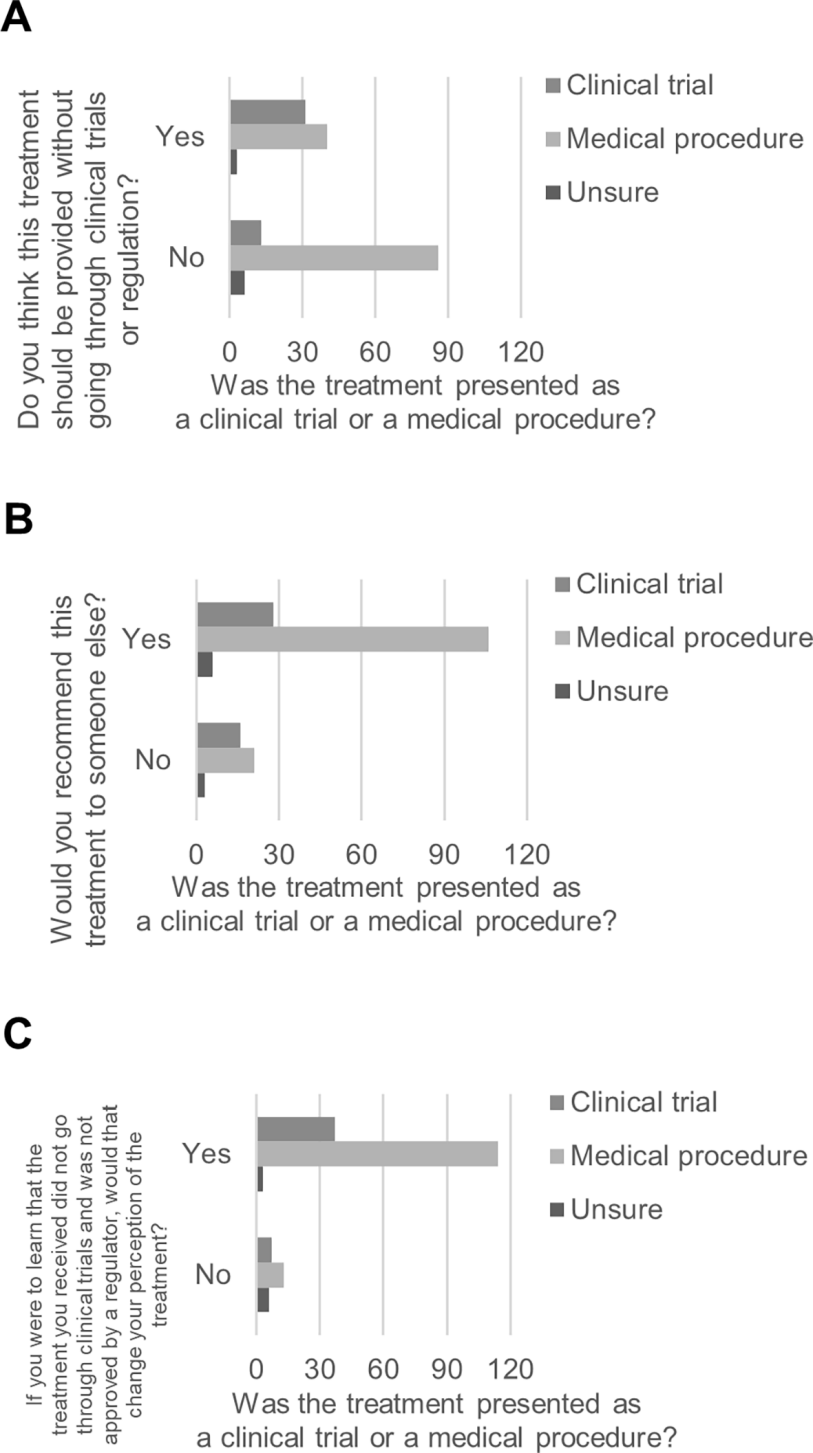
Perspectives on ACBT based on whether participants received it by clinical trial or by medical procedure.

Based on whether they reported having received the ACBT within a clinical trial or as a medical procedure, participants differed in whether they thought the ACBT should be provided without going through clinical trials or regulation, χ ^2^ (2, 179) = 20.40, *p* < .001. Participants who indicated that they received the ACBT as part of a clinical trial were more likely to think that ACBT should be provided without going through clinical trials or regulation (70.5%, *n* = 31) than participants who indicated that they received the treatment procedure as a medical procedure (31.7%, *n* = 40; Figure 1A). The same participants differed in whether they would recommend the ACBT to someone else, χ ^2^ (2, 180) = 8.11, *p* = .017. Participants who indicated that they received the ACBT within a clinical trial were less likely to indicate that they would recommend the ACBT (63.6%, *n* = 28) than participants who indicated that they received the ACBT as a medical procedure (83.5%, *n* = 106; Figure 1B). Based on whether they reported having received the ACBT within a clinical trial or as a medical procedure, participants differed in whether their perception of the ACBT they received would change if they learned that the ACBT did not go through clinical trials and was not approved by a regulator, χ ^2^ (2, 180) = 21.76, *p* < .001. Participants who indicated that they received the ACBT as part of a clinical trial were less likely to indicate that their perception would change (84.1%, *n* = 37) than participants who indicated that they received the ACBT as a medical procedure (89.8%, *n* = 114; Figure 1C).

Non-significant statistical analyses were found between whether the ACBT was presented within a clinical trial or as a medical procedure and what type of ACBT participants received, how participants learned about the ACBT, who the treatment provider was, in what country they received the ACBT, whether or not the treatment provider informed them about any possible side effects of the ACBT, whether and how much they paid for the ACBT, whether the payment for the ACBT was reimbursed or paid out-of-pocket, and whether the evidence for the ACBT was explained.

### Association between study variables depending on whether participants reported having paid and or not paid for the ACBT

Chi-square analyses also revealed that participants differed based on whether or not they paid for the ACBT. Participants’ reports of whether or not they paid for the ACBT varied with the condition for which they sought treatment, χ ^2^ (6, 180) = 13.04, *p* = .042. Among participants who reported that they paid for the ACBT, 81 (48.5%) participants indicated that they sought treatment for a sports injury, 60 (35.9%) participants indicated that they sought treatment for osteoarthritis, 20 (12.0%) sought a cosmetic treatment, and 6 (3.6%) sought other treatments. Among participants who reported that they did not pay for the ACBT, 6 (60.0%) indicated that they sought treatment for osteoarthritis, 3 (30.0%) sought treatment for a sports injury, and 1 (10.0%) sought another treatment.

Participants who paid for the ACBT also differed from participants who did not pay with regards to whether their treatment provider discussed with them how the ACBT is regulated, χ ^2^ (4, 176) = 48.99, *p* < .001. Participants who indicated that they paid for the ACBT were more likely to indicate that the treatment provider discussed with them how the ACBT is regulated (92.0%, *n* = 150) than participants who indicated that they did not pay for the ACBT (30.0%, *n* = 3).

Participants who paid for the ACBT differed from participants who did not pay with regards to whether or not they would recommend the ACBT to someone else, χ ^2^ (2, 180) = 12.59, *p* = .002. Among participants who reported paying for the ACBT, 135 (80.8%) indicated that they would recommend the ACBT, while 4 (40.0%) participants who did not pay for the ACBT agreed with these views.

Statistically non-significant results were found for analyses between whether or not participants paid for the ACBT and the type of ACBT they received, the facility where they received the ACBT, who the treatment provider was, in what country they received the ACBT, whether they thought the ACBT should be provided without going through clinical trials or regulation, and whether their perceptions of the ACBT would change if they learned that the ACBT they received did not go through clinical trials and was not approved by a regulator.

## Discussion

This study surveyed participants who have received ACBT regarding their experiences, knowledge of the regulation and governance of ACBT, and perspectives of the clinical encounter. It explored issues pertaining to pay-to-participate trials, informed consent, and regulatory compliance. Several themes emerged from the data, including the prominent role of healthcare providers throughout the patient journey, informational practices during the clinical encounter, the high prevalence of pay-to-participate trials, patients’ gaps in regulatory knowledge, and patients’ priorities regarding clinical trials and regulation of ACBT.

### The role of healthcare providers

The most commonly reported source through which participants learned about the ACBT was healthcare providers, with other sources such as clinic advertisements and websites, and social media playing a comparatively minor role. Treatment providers were mainly physicians. Our findings highlight the critical role of healthcare providers in providing patients with access to and information about the ACBT. Together, these findings suggest that physicians and healthcare providers play a key role in the patient journey and are at the forefront of the ACBT industry. These findings are consistent with previous studies that report the involvement of physicians in the clinical use and marketing of stem cell therapies ahead of regulatory authorization and supporting evidence. ^42^^,^^43^

### Informational practices during the clinical encounter

Although it is not clear from the survey findings what specific side effects participants were informed about, the finding that the majority of participants were informed by their treatment providers about these side effects is encouraging as it indicates that treatment providers are cognizant of the need to discuss side effects that may be associated with an emerging therapy. The finding that the majority of the participants reported that their treatment providers discussed how ACBTs are regulated and the evidence for the treatment with them strengthens this conclusion. The latter indicates that providers recognize that the evidentiary and regulatory context for ACBT are an important point of emphasis for an emerging therapy that is receiving much social, scientific and regulatory attention and scrutiny. This is supported by our findings that patient perspectives are primarily driven by their beliefs about ACBT. A noteworthy caution is that the study did not assess the quality or accuracy of the information shared and discussed with participants. Medical regulatory authorities may be important for ensuring medical education for healthcare providers on providing and informing patients about ACBT.

### Medical procedures as the primary means of receiving ACBT

The finding that the majority of participants reported that the treatment was presented as a medical procedure rather than a clinical trial is a signal to health product regulators regarding how the industry is operating. Given the push by regulators in some jurisdictions for clinical trials [ Health Canada Policy Position Paper – Autologous Cell Therapy Products] and that the evidentiary and regulatory status of ACBTs remains unclear, it might also serve to alert health products and health professional regulators of the need to work together to ensure that treatment providers are complying with the requirement to conduct clinical trials for treatments that are not established scientifically.

### Reimbursement of payment for ACBT

The finding that some participants received reimbursement for treatment expenses from public and private health insurers is noteworthy as it suggests that insurers view ACBT as beneficial. In maintaining and expanding insurance coverage, care should be taken to align coverage with health technology assessments that weigh the cost of ACBT with its health and economic impacts, even in light of scientific support and regulatory approval. ^44^

### Pay-to-participate trials

Although the majority of participants in the study received ACBT as a medical procedure, a significant number reported having received the ACBT in a clinical trial in which they paid to participate (91.1%, *n* = 41). Of these, all but one participant paid $100 or more (97.6%, *n* = 40) and 21 (51.2%) participants paid out-of-pocket without any reimbursement. Pay-to-participate clinical trials raise a number of ethical concerns, including the skewing of clinical trial funding towards conditions affecting the wealthy, the exploitation of participants’ desire for a treatment, and compromising the scientific integrity of clinical trials. ^45^^–^^47^ These concerns may be acute in the context of ACBT given that the majority of treatment providers are clinicians who may not ordinarily conduct clinical trials and are presumably only doing so mainly for purposes of regulatory compliance. Our findings suggest that regulators ought to be mindful of the ethical issues that may arise from regulatory enforcement initiatives, especially as they impact patient access to care and the conditions under which such care is received.

### Gaps in regulatory knowledge

While participants who received the ACBT as a medical procedure were more likely to think that it should go through clinical trials or receive regulatory approval before being provided, they also indicated that their perception of the treatment would change if they learned it had not gone through clinical trials or received regulatory approval. These responses suggest an assumption among participants that ACBT provided as medical procedures are under the purview of a regulatory body. Participants expressed a wide variability in what type of regulatory body they thought was responsible for regulating ACBT, with only a minority of them (21.4%, *n* = 77) correctly indicating that a federal regulatory authority (Health Canada and/or the US FDA) was responsible. Designing the regulatory system to be consistent with patients’ expectations of regulatory oversight therefore represents an important step in maintaining the trust of patients in ACBT.

### Improving clinical trials and regulations

Safety emerged as a major theme of concern among participants, suggesting a public expectation for regulatory initiatives to prioritize safety. Regulations that address safety concerns may therefore be more likely to receive public support.

### Limitations

While the use of an anonymous online survey had several advantages, including quick data collection from a broad sample of patients and minimization of self-reporting bias, these methods also limited our certainty in the authenticity of responses. The use of lottery draws may incentivize invalid responses for monetary gain. To minimize these risks, we removed any likely bot or duplicate responses from our analyses.

### Conclusion and future directions

The study makes a novel contribution to the literature by providing the first analysis of patients’ experiences and perceptions of an emerging cell-based therapy within an evolving regulatory landscape. The findings serve as a valuable resource for developing policy, promoting scientific rigor, and ensuring ethical oversight of ACBT and other upcoming cell-based therapies. The study also highlights participants’ prioritization of the safety of ACBT while identifying critical gaps in their regulatory knowledge. Their experiences, knowledge, and perspectives provide critical insights towards the advancement of regulations pertaining to ACBT in Canada and the U.S. The important role of healthcare providers in how participants are informed about and receive ACBT warrants the involvement of provincial and state medical regulatory authorities in the regulation and enforcement of standards around informed consent, particularly among ACBT that are being provided as medical procedures. Conducting research on healthcare providers to explore their informed consent processes and pricing structure more thoroughly for ACBT may support recommendations for their regulation. To this end, our team is currently conducting a complementary study based on interviews with clinicians and clinical researchers who have sponsored ACBT in clinical trials in Canada and Australia. Our amplification of the patient voice by assessing their experiences, knowledge, and perspectives contributes a patient-centred perspective to the advancement of regulations pertaining to ACBT.

## Data Availability

Open Science Framework: Clinical Use of Autologous Cell-Based Therapies in an Evolving Regulatory Landscape: A Survey of Patient Experiences and Perceptions (Dataset and Survey Questionnaire), https://doi.org/10.17605/OSF.IO/7RSJX. ^48^ This project contains the following underlying data:
- S2 Appendix - Dataset.xlsx Open Science Framework: Clinical Use of Autologous Cell-Based Therapies in an Evolving Regulatory Landscape: A Survey of Patient Experiences and Perceptions (Dataset and Survey Questionnaire), https://doi.org/10.17605/OSF.IO/7RSJX. ^48^ This project contains the following extended data:
- S1 Appendix - Survey.pdf Data are available under the terms of the Creative Commons Attribution 4.0 International license (CC-BY 4.0).
