# Supplementary material for: Clinical use of autologous cell-based therapies in an evolving regulatory landscape: A survey of patient experiences and perceptions
Source: F1000Res. 2024 Aug 27;12:1165. Originally published 2023 Sep 18. [Version 2] doi: 10.12688/f1000research.141002.2 (PMC11489844; doi:10.12688/f1000research.141002.2)
Supplement: Peer review history

# Reviewer report: Reviewer response for version 2

**DOI:** 10.5256/f1000research.170536.r317354

**Published:** 2024-10-18

**Authors:** Yann Joly

**Affiliations:** 1 McGill University, Montreal, QC, Canada

I accept this manuscript without reservation for indexing.

Is the work clearly and accurately presented and does it cite the current literature?

Yes

If applicable, is the statistical analysis and its interpretation appropriate?

Yes

Are all the source data underlying the results available to ensure full reproducibility?

Yes

Is the study design appropriate and is the work technically sound?

Partly

Are the conclusions drawn adequately supported by the results?

Yes

Are sufficient details of methods and analysis provided to allow replication by others?

Partly

Reviewer Expertise:

NA

I confirm that I have read this submission and believe that I have an appropriate level of expertise to confirm that it is of an acceptable scientific standard.

# Reviewer report: Reviewer response for version 2

**DOI:** 10.5256/f1000research.170536.r317355

**Published:** 2024-09-10

**Authors:** Li Du

**Affiliations:** 1 University of Macau, Macau, China

I would like to express my thanks for the authors' explanations and revisions. I am satisfied with this revised version.

Is the work clearly and accurately presented and does it cite the current literature?

Yes

If applicable, is the statistical analysis and its interpretation appropriate?

Partly

Are all the source data underlying the results available to ensure full reproducibility?

Yes

Is the study design appropriate and is the work technically sound?

Yes

Are the conclusions drawn adequately supported by the results?

Yes

Are sufficient details of methods and analysis provided to allow replication by others?

Yes

Reviewer Expertise:

NA

I confirm that I have read this submission and believe that I have an appropriate level of expertise to confirm that it is of an acceptable scientific standard.

# Reviewer report: Reviewer response for version 1

**DOI:** 10.5256/f1000research.154413.r292492

**Published:** 2024-07-03

**Authors:** Yann Joly

**Affiliations:** 1 McGill University, Montreal, QC, Canada

This manuscript presents an interesting study of patient experiences and perceptions of autologous cell-based therapy (ACBT) procedures. The piece is clear and well written it is informative of the type of patients interested in these procedures in North America, and about the procedures themselves. The context information provided, while succinct, is recent and accurate.

 It is surprising to me that the authors did not choose to collect a minimal set of demographic information about their respondents, age range, sex, education level, for example, which would have provided a much richer dataset. In the same vein, it is unclear whether they retained the information about countries of origin (U.S., Canada). If they did, it was not included in the analysis parameters they used. Given that the survey was anonymous, were the authors able to filter out potential duplicates? (Participants that may have responded twice or more to the questionnaire). Were respondents compensated for their responses or was the contribution non remunerated? I would have also liked to have more information on whether the quantitative data obtained was statistically significant or sufficiently representative of the U.S./Canadian population of patients seeking ACBT.  This seems unlikely and it would be important to disclose this study limitation.

 In the discussion section, the authors make the most of their data providing interesting interpretations and inferences for their results. I have some reserve about the positive inference made by the authors about international practices during the clinical encounter. The fact that service providers participating in a generally fraudulent practice, can speak to some of its basic regulatory and mechanistic concepts with customers is hardly reassuring, it simply makes their otherwise false representations more believable to them. The findings about the (growing?) importance of the pay-to-participate type of stem cell clinical trials and, that of reimbursement payments for ACBT procedures are much more interesting and warrant further exploration.

 In sum a good short manuscript, to keep up with the fraudulent stem cell businesses ethical challenge.  The study itself make for a good pilot that could be further developed to be used more widely. If so, please make sure to collect some demographic data with the responses. This manuscript presents an interesting study of patient experiences and perceptions of autologous cell-based therapy (ACBT) procedures. The piece is clear and well written it is informative of the type of patients interested in these procedures in North America, and about the procedures themselves. The context information provided, while succinct, is recent and accurate.

 It is surprising to me that the authors did not choose to collect a minimal set of demographic information about their respondents, age range, sex, education level, for example, which would have provided a much richer dataset. In the same vein, it is unclear whether they retained the information about countries of origin (U.S., Canada). If they did, it was not included in the analysis parameters they used. Given that the survey was anonymous, were the authors able to filter out potential duplicates? (Participants that may have responded twice or more to the questionnaire). Were respondents compensated for their responses or was the contribution non remunerated? I would have also liked to have more information on whether the quantitative data obtained was statistically significant or sufficiently representative of the U.S./Canadian population of patients seeking ACBT.  This seems unlikely and it would be important to disclose this study limitation.

 In the discussion section, the authors make the most of their data providing interesting interpretations and inferences for their results. I have some reserve about the positive inference made by the authors about international practices during the clinical encounter. The fact that service providers participating in a generally fraudulent practice, can speak to some of its basic regulatory and mechanistic concepts with customers is hardly reassuring, it simply makes their otherwise false representations more believable to them. The findings about the (growing?) importance of the pay-to-participate type of stem cell clinical trials and, that of reimbursement payments for ACBT procedures are much more interesting and warrant further exploration.

 In sum a good short manuscript, to keep up with the fraudulent stem cell businesses ethical challenge.  The study itself make for a good pilot that could be further developed to be used more widely. If so, please make sure to collect some demographic data with the responses.

Is the work clearly and accurately presented and does it cite the current literature?

Yes

If applicable, is the statistical analysis and its interpretation appropriate?

Yes

Are all the source data underlying the results available to ensure full reproducibility?

Yes

Is the study design appropriate and is the work technically sound?

Partly

Are the conclusions drawn adequately supported by the results?

Yes

Are sufficient details of methods and analysis provided to allow replication by others?

Partly

Reviewer Expertise:

Law, bioethics and public policy in modern medicine

I confirm that I have read this submission and believe that I have an appropriate level of expertise to confirm that it is of an acceptable scientific standard, however I have significant reservations, as outlined above.

# Response

**Published:** 2024-08-16

**Authors:** Ubaka Ogbogu

**Affiliations:** Law, University of Alberta, Edmonton, Alberta, Canada

**Reviewer:** This manuscript presents an interesting study of patient experiences and perceptions of autologous cell-based therapy (ACBT) procedures. The piece is clear and well written it is informative of the type of patients interested in these procedures in North America, and about the procedures themselves. The context information provided, while succinct, is recent and accurate.

 It is surprising to me that the authors did not choose to collect a minimal set of demographic information about their respondents, age range, sex, education level, for example, which would have provided a much richer dataset.

**Response:** We initially considered collecting demographic information but decided against it for two main reasons. First, to minimize the risk of “survey fatigue” and to improve response rates and data quality, we shortened the questionnaire to focus solely on questions directly related to our research objectives. Survey fatigue is a well-documented issue, particularly during the COVID-19 pandemic [1-5]. Second, while demographic data could provide insights into how demographic variables influence perceptions, we determined that our research questions could be answered without it. Additionally, our analysis did not rely on demographic factors or treat the responses as representative of any specific population of patients seeking ACBT. However, we acknowledge the reviewer’s point and will incorporate demographic information in future studies on this topic.

**Reviewer:** In the same vein, it is unclear whether they retained the information about countries of origin (U.S., Canada). If they did, it was not included in the analysis parameters they used.

**Response:** We did not collect information regarding the country of origin, as it was not necessary for answering our research question. However, we did gather data on the country where respondents received ACBT, which is detailed in the “Results” section under “Part 1: Summary of Participants’ Experiences.”

**Reviewer:** Given that the survey was anonymous, were the authors able to filter out potential duplicates? (Participants that may have responded twice or more to the questionnaire).

**Response:** Yes, duplicates were filtered out by Qualtrics and through manual screening. We have already mentioned this process in the Methods section of the paper. Additionally, we noted in the Limitations section that “we removed any likely bot or duplicate responses from our analysis.”

**Reviewer:** Were respondents compensated for their responses or was the contribution non remunerated? 

**Response:** Survey respondents were entered into a lottery draw for a chance to win a $50 CAD gift card. This incentive was approved by the University of Alberta Research Ethics Board. One respondent received the incentive. We already noted the lottery draw in the Limitations section, but we have also updated the Methods section to clearly note the incentive.

**Reviewer:** I would have also liked to have more information on whether the quantitative data obtained was statistically significant or sufficiently representative of the U.S./Canadian population of patients seeking ACBT.  This seems unlikely and it would be important to disclose this study limitation.

**Response:** We have included a statement in the Limitations section to address this comment. The statement reads: “The quantitative data reported in this study is not sufficiently representative of the population of patients seeking ACBT in the U.S. and Canada. Therefore, the data should not be interpreted as generalizable to this population.”

**Reviewer:** In the discussion section, the authors make the most of their data providing interesting interpretations and inferences for their results. I have some reserve about the positive inference made by the authors about international practices during the clinical encounter. The fact that service providers participating in a generally fraudulent practice, can speak to some of its basic regulatory and mechanistic concepts with customers is hardly reassuring, it simply makes their otherwise false representations more believable to them.

**Response:** We are grateful to the reviewer for this observation. We have added a statement in the Discussion section to acknowledge the comment.

**Reviewer:** The findings about the (growing?) importance of the pay-to-participate type of stem cell clinical trials and, that of reimbursement payments for ACBT procedures are much more interesting and warrant further exploration.

**Response:** We are grateful to the reviewer for this observation and will keep it in mind for further exploration.

**Reviewer:** In sum a good short manuscript, to keep up with the fraudulent stem cell businesses ethical challenge.  The study itself make for a good pilot that could be further developed to be used more widely. If so, please make sure to collect some demographic data with the responses.

**Response:** Noted. We will endeavour to address this comment in future studies. Thank you! 

**References**

1. Lavrakas, P. J. (2008). Respondent fatigue. In Encyclopedia of Survey Research Methods (pp. 743-743). SAGE Publications, Inc., https://doi.org/10.4135/9781412963947.
2. Galesic, M., & Bosnjak, M. (2009). Effects of Questionnaire Length on Participation and Indicators of Response Quality in a Web Survey. *The Public Opinion Quarterly*, *73*(2), 349–360. http://www.jstor.org/stable/25548084
3. Herzog, A. R., & Bachman, J. G. (1981). Effects of Questionnaire Length on Response Quality. *The Public Opinion Quarterly*, *45*(4), 549–559. http://www.jstor.org/stable/2748903
4. Jeong, D., Aggarwal, S., Robinson, J., Kumar, N., Spearot, A., & Park, D. S. (2023). Exhaustive or exhausting? Evidence on respondent fatigue in long surveys. *Journal of Development Economics*, *161*, 102992.
5. De Koning, R., Egiz, A., Kotecha, J., Ciuculete, A. C., Ooi, S. Z. Y., Bankole, N. D. A., ... & Kanmounye, U. S. (2021). Survey fatigue during the COVID-19 pandemic: an analysis of neurosurgery survey response rates. *Frontiers in surgery*, *8*, 690680.

# Reviewer report: Reviewer response for version 1

**DOI:** 10.5256/f1000research.154413.r223427

**Published:** 2023-12-15

**Authors:** Li Du

**Affiliations:** 1 University of Macau, Macau, China

The research is well-structured and written. It offers a unique viewpoint from patients who underwent the ACBT about their experience and comprehension of ACBT regulation’s legal aspects. I recommend accepting it for publication. However, I have two concerns regarding the discussion section where the authors discuss payment reimbursement for ACBT and improvements for clinical trials and regulations.

 The authors’ assertion that insurers perceive ACBT as beneficial in the reimbursement section does not appear well-supported by the results. More explanation is needed to support this point. For improving regulations, safety is typically a fundamental principle for clinical trial regulations. Authors should further elaborate on this issue, explaining why the authors raised this concern. For example, are there regulations that place safety to a secondary role, or have certain regulations been improperly interpreted or enforced, thereby diminishing the importance of safety in favor of other considerations?

Is the work clearly and accurately presented and does it cite the current literature?

Yes

If applicable, is the statistical analysis and its interpretation appropriate?

Partly

Are all the source data underlying the results available to ensure full reproducibility?

Yes

Is the study design appropriate and is the work technically sound?

Yes

Are the conclusions drawn adequately supported by the results?

Yes

Are sufficient details of methods and analysis provided to allow replication by others?

Yes

Reviewer Expertise:

biotechnology law and policy

I confirm that I have read this submission and believe that I have an appropriate level of expertise to confirm that it is of an acceptable scientific standard, however I have significant reservations, as outlined above.

# Response

**Published:** 2024-01-25

**Authors:** Ubaka Ogbogu

**Affiliations:** Law, University of Alberta, Edmonton, Alberta, Canada

We are grateful to the reviewer for the positive feedback and for the comments on the article. We have provided responses addressing the reviewer's comments below, as follows:

 Reviewer comment: The authors’ assertion that insurers perceive ACBT as beneficial in the reimbursement section does not appear well-supported by the results. More explanation is needed to support this point.

 Our response: Thank you for this feedback. In the discussion section we suggested that insurers view ACBT as beneficial based on the finding that some participants are receiving reimbursement for treatment expenses from public and private health insurers. Generally, a criterion that health insurers (and health technology assessment reviews that inform reimbursement decisions) consider in deciding whether to reimburse the cost of a health intervention or drug is whether it provides a clinical benefit that is superior or comparable to existing treatment options (CADTH 2020; Nagase et al 2019). While it is not the only criteria (others include survival gains, cost and cost-effectiveness, clinical safety, improvement in surrogate outcomes, etc.), we use the notion of "beneficial" to link the finding that insurers are reimbursing ACBT costs to the notion that they do so because they believe, among other things, that it offers relative clinical benefit. We hope this explanation clarifies. 

 Reviewer comment: For improving regulations, safety is typically a fundamental principle for clinical trial regulations. Authors should further elaborate on this issue, explaining why the authors raised this concern. For example, are there regulations that place safety to a secondary role, or have certain regulations been improperly interpreted or enforced, thereby diminishing the importance of safety in favor of other considerations?

 Our response: We have not raised the issue of safety as a "concern" in the discussion. Rather, we discussed the finding that safety emerged as a major theme of concern for the participants in the study (so, it was raised by the participants, not us) and we then reflected that this suggests a need to prioritize and address those safety concerns through future regulations. However, we note, in agreement with the reviewer, that it may be necessary to examine the extent to which extant regulations advance or fail to advance safety in the context of clinical trials of ACBT, and how safety regulations operate in practice or are perceived. This work, though important, is outside the scope of the current study. We are presently conducting other empirical studies with regulators and ACBT trialists where we examine how extant regulations, including safety regulations, shape and influence clinical trials and clinical uses of ACBT, and their perceptions of such regulations. We believe the results of the in progress study will answer some of the questions posed by the reviewer regarding the role that safety regulations play, and how they are perceived, within the context of ACBT clinical trials. 

 Citations:

 CADTH (Canada's Drug and Health Technology Agency), Procedures for CADTH Reimbursement Reviews, January 2024, https://www.cadth.ca/sites/default/files/Drug_Review_Process/CADTH_Drug_Reimbursement_Review_Procedures.pdf

 Nagase, F.N.I., Stafinski, T., Sun, J. *et al.* Factors associated with positive and negative recommendations for cancer and non-cancer drugs for rare diseases in Canada. *Orphanet J Rare Dis* **14**, 127 (2019). https://doi.org/10.1186/s13023-019-1104-7
